# Adolescent Ethanol Exposure Enhances NMDA Receptor-Mediated Currents in Hippocampal Neurons: Reversal by Gabapentin

**DOI:** 10.1038/s41598-017-12956-6

**Published:** 2017-10-13

**Authors:** H. S. Swartzwelder, Maeng-Hee Park, Shawn Acheson

**Affiliations:** 1 Neurobiology Research Laboratory, Durham VA Medical Center, Durham, N.C 27705 USA; 2 Department of Psychiatry and Behavioral Sciences, Duke University Medical Center, Durham, N.C 27710 USA

## Abstract

Adolescent intermittent ethanol (AIE) exposure compromises neural function into adulthood. We have reported that astrocyte-secreted thrombospondins, and their target neuronal receptors (α2δ−1) are upregulated in the hippocampus in adulthood after AIE, suggesting aberrant excitatory synaptogenesis and hyperexcitability in memory-related circuits. Gabapentin antagonizes the interaction of thrombospondins (TSPs) with the α2δ−1 receptor, and thus may reverse or ameliorate the effects of AIE on hippocampal function. Adolescent rats were exposed to AIE or vehicle. In adulthood, hippocampal slices were prepared. Half of the slices from each animal were pre-incubated in normal artificial cerebrospinal fluid (aCSF) while half were pre-incubated in aCSF containing gabapentin. Whole-cell voltage clamp recordings were then made from CA1 pyramidal cells in normal aCSF. Evoked, N-methyl-D-aspartate (NMDA) receptor-mediated currents were recorded at baseline, and after application of the GluN2B antagonist, RO25–6981. Current amplitudes were higher in neurons from AIE-exposed animals. However, no amplitude increase was observed in neurons from slices that had been pre-incubation in gabapentin. GluN2B antagonism reduced NMDA receptor-mediated currents more efficaciously in cells from AIE-exposed animals, an effect that was also reversed by pre-incubation in gabapentin. These findings identify a mechanism underlying the enduring effects of AIE, and a clinically-utilized agent that may ameliorate those effects.

## Introduction

Alcohol is the world’s most widely used recreational drug, and most people in the U.S. begin use during adolescence or young adulthood. National surveys show that 77% of 12th graders and 85% of college students have tried alcohol. In addition, 29% of 12th graders and 42% of college students report having had five or more drinks in a row during the last two weeks^1^. This prevalence of heavy drinking occurs during a period when the brain is undergoing rapid changes in structure and function that make it vulnerable to negative consequences of alcohol exposure^2–5^.

The enduring effects of ethanol exposure during adolescence and young adulthood have recently become the subject of intense investigation in both humans and animal models. In both humans and rodents, adolescents and young adults manifest differential responsiveness to acute ethanol^6–10^, are sensitive to enduring negative effects of repeated ethanol exposure that persist into adulthood (see^11,12^ for reviews), and it is well known that drinking onset at young ages is strongly associated with alcohol abuse in adulthood^13,14^. Thus, it is clear that adolescence represents a period of distinctive sensitivity to the enduring effects of repeated ethanol exposure. However, the neural mechanisms underlying that sensitivity are just beginning to be investigated.

We have recently reported elevations of astrocyte-secreted thrombospondins (TSPs) in hippocampal area CA1 25 days after adolescent intermittent ethanol (AIE) exposure in rats^15^, as well as lowered thresholds for the induction of long-term potentiation (LTP) in that region^16^. This is noteworthy because certain TSPs are known to promote the genesis of excitatory synapses, which could underlie hyperexcitability. In normal development, TSP-2 levels (which we have observed elevated after AIE) are maximal during the first 10 postnatal days in the rat, and then decrease by postnatal day 20 and remain low thereafter. That TSP-2 is increased by AIE suggests that the ethanol exposure may induce a period of aberrant excitatory synaptogenesis that persists into adulthood. Importantly, we have also observed an AIE-induced increase in the α2δ−1 subunit of the L-type calcium channel complex^15^, with which TSPs interact to initiate synaptogenesis – an interaction that is directly antagonized by gabapentin (Neurontin)^17^. Combined with the increased LTP induction by mild stimulus trains and neuronal loss in hippocampal area CA1 after AIE^16^, these findings suggest that AIE may induce a state of elevated susceptibility to hyperexcitability and possible liability to excitotoxic cell loss, possibly through unscheduled astrocyte-mediated excitatory synaptogenesis.

The antagonism of thrombospondin-induced synaptogenesis by gabapentin^17^, suggests the possibility that the excitatory effects of AIE in hippocampal area CA1 could be mediated by glutamatergic receptors and antagonized by gabapentin. Because the NMDA subtype of glutamate receptors is associated with both synaptic plasticity and excitotoxicity^18^, we designed the present experiments to assess the effects of AIE on the amplitude NMDA receptor-mediated currents in CA1 pyramidal cells. We hypothesized that AIE would increase the amplitude of those currents and that pre-treatment of hippocampal slices with gabapentin would antagonize that effect of AIE.

## Methods

The procedures in this study were conducted in accordance with the guidelines of the American Association for the Accreditation of Laboratory Animal Care and the National Research Council’s Guide for Care and Use of Laboratory Animals. In addition, they were approved by the Durham VA Medical Center and the Duke University Animal Care and Use Committees.

### Adolescent Intermittent Ethanol Exposure

Twenty-eight male, Sprague-Dawley rats (Charles River, USA) were double-housed in a temperature- and humidity-controlled room. They had ad libitum access to food and water. They were dosed using methods previously described in Risher *et al*.^15^. The rats were delivered at PND-25 and allowed to acclimatize for 5 days on a reverse 12:12-hr light:dark cycle (lights off at 9:00 am) prior to beginning AIE or distilled water (AIW) exposure on PND-30, which consisted of 10 doses of 5 g/kg ethanol (35% v/v in dH_2_O at 18.12 mL/kg, VWR, Suwanee, GA, USA) or isovolumetric dH_2_O administered by intragastric gavage using a 2 days on, 1 day off, 2 days on, 2 days off schedule for 16 days. This was followed by a 25-day period of no treatment, allowing the animals to reach adulthood prior to sacrifice. The ethanol dose was selected in order to produce blood ethanol concentrations (BECs) consistent with adolescent human BECs during binge drinking episodes^19^. We have found that rats of this age, sex, and strain, receiving 5 g/kg ethanol (i.g.), achieved mean blood ethanol concentrations of 199.7 mg/dl (±19.9) 60 minutes after the first dose, and 172.8 (±13.3) 60 minutes after the last dose^15^. The rats in that study were treated exactly as those in the present experiment. Moreover, those blood ethanol concentrations are consistent with those achieved in our earlier studies^20^.

### Electrophysiological Recording

The rats were decapitated after being anesthetized with isoflurane. The brains were removed and placed in 4 °C artificial cerebrospinal fluid (aCSF) containing (in mM): 120 NaCl, 3.3 KCl, 1.23 NaH2PO_4_, 26 NaHCO_3_, 1.2 MgSO_4_, 1.8 CaCl_2_ and 10 D-Glucose at pH 7.3, previously saturated with 95%O_2_/5%CO_2_. Hippocampal slices (300 μm thick) were cut on a vibratome (100PLUS, Sectioning System, Ted Pella, Inc., Redding CA) and incubated in a holding chamber containing aCSF continuously infused with 95% O_2_/5% CO_2_ at 20–24 °C. Half of the slices from each animal were pre-incubated for one hour in normal aCSF and half were pre-incubated for one hour in aCSF containing 30 μM gabapentin. Ten slices from separate control animals were used for experiments in the absence of gabapentin pre-treatment, and 11 slices were used for experiments after gabapentin pre-treatment. Eight slices from separate AIE animals were used for experiments in the absence of gabapentin pre-treatment, and nine slices were used for experiments after gabapentin pre-treatment.

Patch pipettes (borosilicate glass capillary tubing (1.5 mm O.D., 1.05 mm I.D., World Precision Instruments, Sarasota, FL) were pulled on a Flaming-Brown horizontal microelectrode puller (Model P-97, Sutter Instrument Co, Novato, CA), and were filled with an intracellular solution containing (in mM): 130 Cs-gluconate, 7 CsCl, 10 HEPES, 4 Mg-ATP, 0.5 Tris-GTP (pH = 7.25). The lidocaine derivative QX-314 (4.0 mM)(Sigma Chemical Co., St Louis, MO) was included to suppress fast sodium currents, and GABA_B_ receptor-mediated currents. In certain experiments, Alexa Fluor-568 (50–80 μM, Molecular Probes, Carlsbad, CA) was also included in the internal solution. Osmolarity was adjusted to 285 mOsm. The pipette resistances were between 4–7 Mohm.

After one hour of incubation, slices were transferred into a submersion recording chamber and secured in place with a piece of platinum wire. Chamber temperature was maintained at 34 °C during recording. Whole cell voltage clamp recordings were made from CA1 pyramidal cells visualized on a Zeiss microscope equipped with infrared differential interference contrast optics and a 40x water immersion objective (Zeiss, Oberkochen, Germany). After the establishment of whole-cell recording preparation, stable and long-lasting tight-seal recordings were achieved in most instances. Evoked, NMDA receptor-mediated EPSCs (eEPSCs) were recorded with an Axopatch 200B amplifier (Molecular Devices, Union City, CA). Output current signals were DC-coupled to a digital storage oscilloscope (TDS 2014, Tektronix, Inc., Beaverton, OR). Series resistance was monitored throughout the recording period and cells were not used if the series resistance changed by more than 20%. Digitized data were also acquired and stored using Strathclyde Electrophysiology Software, specifically the Whole Cell Program (WINWCP) with an interface (BNC-2090, National Instruments, Austin, TX) to a PC-computer. Real–time measurements of the eEPSC amplitude were made and displayed simultaneously on a second PC computer using a custom-written program developed with Labview (Version 6i, National Instrument) by Dr. Maeng-Hee Kang-Park.

**Figure 1:**
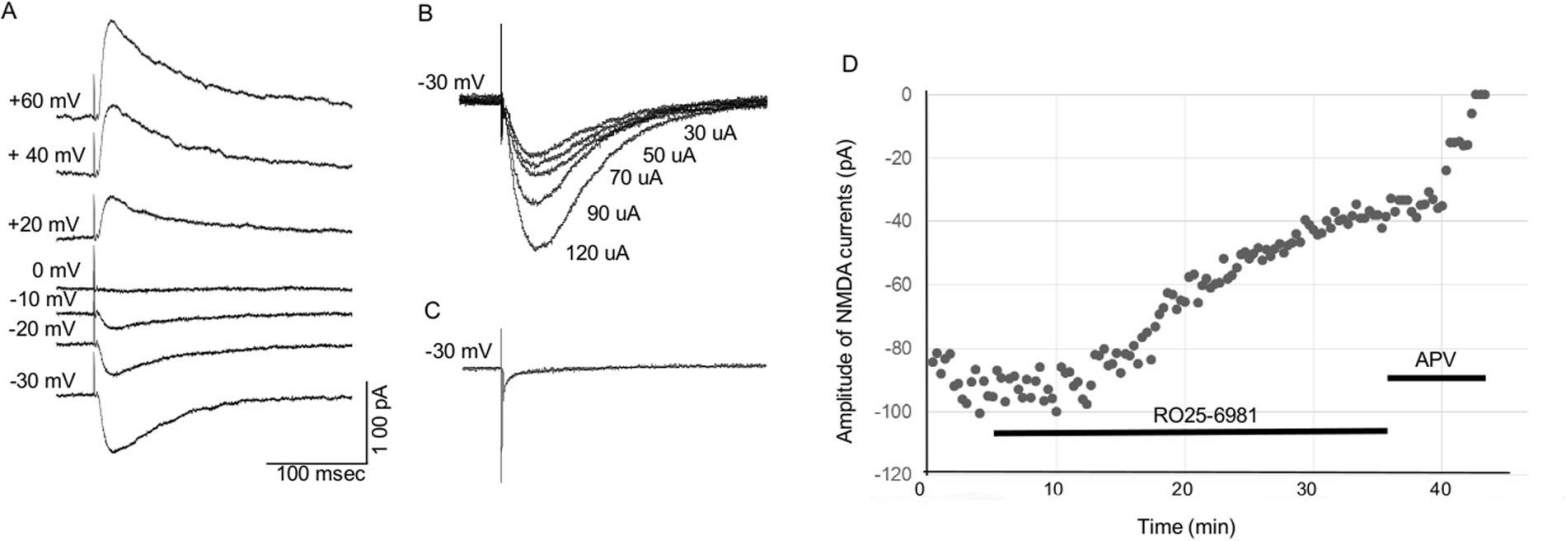
NMDA Receptor-Mediated Currents Recorded from CA1 Pyramidal Cells. (**A**) Currents recorded at a range of holding potentials to assess voltage dependency. Subsequent currents for analysis were recorded at −30 mV. (**B**) Input/output function for NMDA current in a representative CA1 pyramidal cell across stimulus intensities of 30–120 μA. (**C**). The application of 40 μM d-APV eliminated the recorded currents, establishing their NMDA receptor dependency. (**D**) Dot-plot illustrating the effects of RO25–6981 (1 uM) and d-APV (40 μM) on NMDA receptor-mediated currents. Each dot represents the amplitude of one eEPSC recorded from a representative control neuron used in the present experiment. Note that the reduction of current by RO25–6981 is approximately 60% in this cell, consistent with the average reduction that we observed among all control neurons.

In the presence of the GABA_A_ receptor antagonist, bicuculline (100 μM), and the a-amino-3-hydroxy-5-methylisoxazole-4-propionic acid (AMPA) receptor antagonist, 6,7-dinitroquinoxaline-2,3-dione (DNQX)(20 μM), NMDA receptor-mediated EPSCs were evoked at a holding potential of −30 mV by electrical stimulation through a concentric bipolar tungsten electrode (A-M systems, Inc., Carlsborg, WA) placed into the CA3-CA1 transition zone in order to record from CA1 pyramidal cells after activation of Schaffer collateral fibers. The voltage dependency of evoked currents was assessed in voltage clamp mode by holding cells at a range of holding potentials between −50 mV and +60 mV, and currents reversed near 0 mV (see Fig. 1A for illustration). The response threshold was first determined by increasing the intensity of constant current rectangular wave pulses generated by an isolated stimulator (Grass S88, Grass Instrument CO, Quincy, MA) until detectable responses occurred. Input-output curves were generated for each cell using stimulus pulses of 50 μA–500 μA (0.1 ms duration), and a stimulus intensity that evoked 50% maximal response used to evoke baseline eEPSCs for each cell. This stimulus intensity fell within the 100–200 μA range across cells.

NMDA receptor-mediated eEPSCs were evoked at an inter-stimulus interval of 0.033 Hz, and recorded for at least 20 minutes in order to establish a stable baseline. After the baseline was established the GluN2B antagonist, Ro 25–6981 (1 μM), was applied to each slice in order to determine the percentage of the recorded currents that were driven by that receptor subtype. After eEPSCs were sampled in the presence of Ro 25–6981 for 30 minutes, 40 μM D-(−)-2-Amino-5-phosphonopentanoic acid (d-APV) was applied to assure that the currents were carried exclusively by NMDA receptors. Data from one cell from each animal were used for statistical analyses, and the data points for each cell represent the average of ten sequential eEPSCs recorded prior to, and during presence of R025–6981.

### Statistical Analyses

To test our initial hypothesis, that AIE leads to increased eEPSC amplitudes and this increase is largely GluN2B dependent, we employed a 2 × 2 Analysis of Variance (ANOVA) using ethanol pre-treatment (AIW vs. AIE) as a between subjects independent variable (IV) and RO25–6981 as a repeated measures IV. The latter was treated as a repeated measure because eEPSCs were recorded before and after application of RO25–6981. Data analyzed in this initial analysis were drawn from slices pre-incubated in normal aCSF (without 30 μM gabapentin). Based on our hypotheses, we anticipated a statistically significant interaction with simple main effects of AIE before but not after application of RO25–6981.

Our second hypothesis, that gabapentin pre-incubation would reduce the effect of AIE on eEPSC amplitude, was assessed using an identical analytic strategy for slices that had been pre-incubated in 30 μM gabapentin prior to recording. As above, we employed a 2 × 2 repeated measures ANOVA (RM-ANOVA) with AIE as a between subjects IV and RO25–6981 as the repeated measure IV. Based on our hypotheses, we expected gabapentin pre-incubation to attenuate or eliminate the interaction between AIE and RO25–6981.

To better understand the effect of gabapentin directly, we also calculated the percent decrease in eEPSCs induced by RO25–6981, and subjected those data to a 2 × 2 RM-ANOVA using AIE as a between subjects IV and gabapentin as a repeated measure IV. The latter variable was treated as a repeated measure because slices pre-incubated in standard aCSF and aCSF+ gabapentin were taken from the same animal. Based on our hypotheses, we expected an interaction to demonstrate that the RO25–6981-induced decrease in eEPSCs would be diminished by gabapentin pre-incubation in AIE but not AIW slices.

Within each of the analyses described above, the interaction serves as the basis of our hypotheses. As such, main effects are reported as secondary outcomes. Where interaction effects were statistically significant, simple main effects analyses were conducted using standard parametric statistics following significant interactions. Alpha was set to 0.05 for all analyses.

## Results

Evoked NMDA receptor-mediated currents were readily recorded from CA1 pyramidal cells in all slices. The voltage sensitivity and input/output functions of a representative neuron are shown in Fig. 1A,B, and were consistent with NMDA receptor-mediated currents recorded in our previous studies (see^21^). Bath application of APV eliminated the currents, indicating their NMDA receptor-mediated specificity (see Fig. 1C,D).

**Figure 2:**
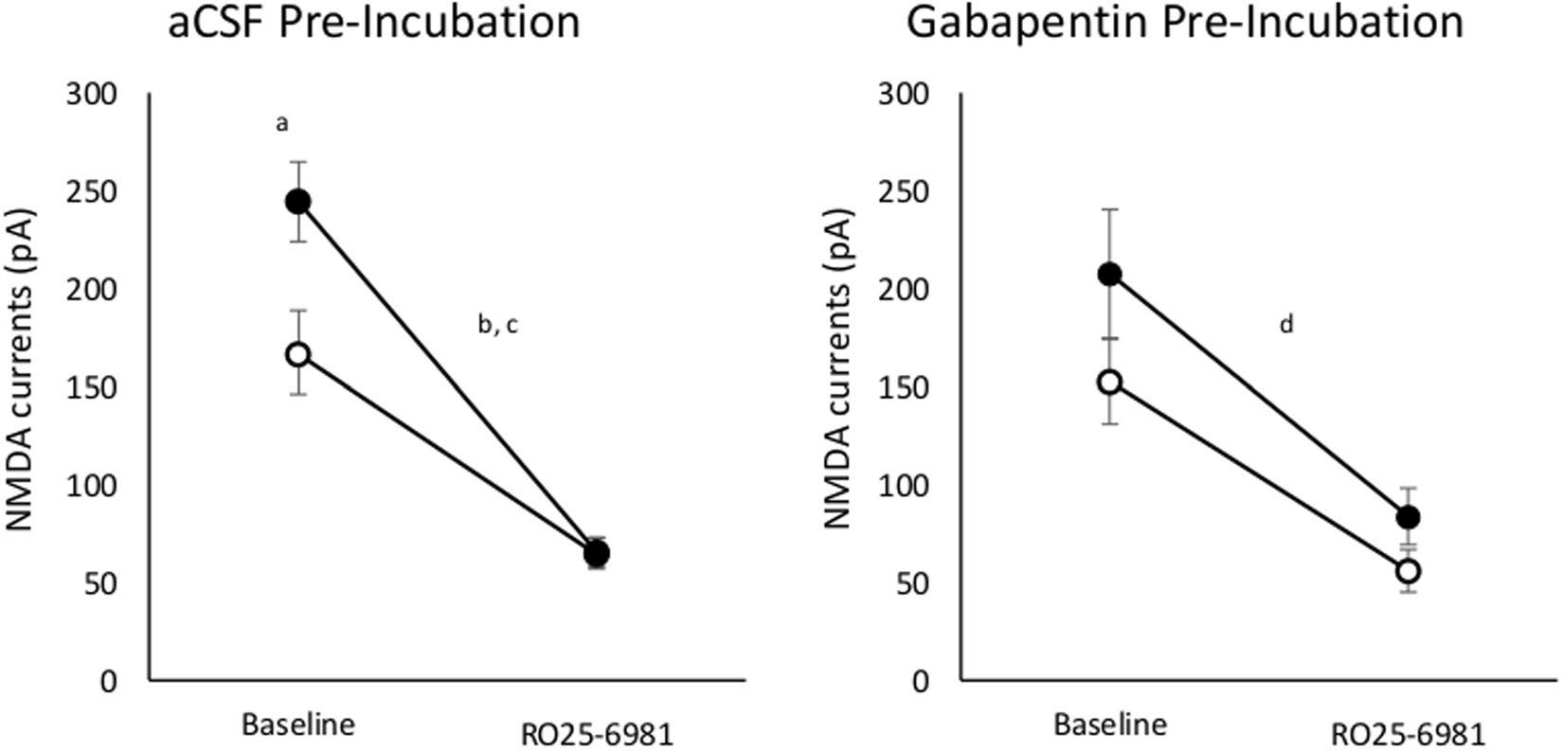
Mean (±SEM) amplitude of NMDA receptor-mediated currents recorded before and after bath application of 1 μM RO25–6981 in slices pre-incubated in aCSF (left panel) or aCSF+ 30 μM gabapentin (right panel) in CA1 pyramidal cells in slices from AIW animals (open circles) and AIE animals (closed circles). ^a^In the absence of gabapentin pre-incubation, current amplitudes in cells from AIE animals were higher than those in cells from AIW animals (p < 0.05) prior to application of RO25–6981. Following application of RO25–6981, current amplitudes were significantly reduced in cells from both AIW^b^ and AIE^c^ animals (p < 0.05). ^d^There was a similar main effect of RO25–6981 in slices that were pre-incubated in gabapentin (p < 0.05).

Consistent with our initial hypotheses, CA1 pyramidal cells recorded in slices from AIE exposed animals generated NMDA receptor-mediated eEPSCs of greater amplitude than did neurons from control animals, and these were more potently inhibited by RO25–6981 in slices from AIE exposed animals than in those from AIW animals. Consistent with our hypothesis, there was a statistically significant interaction of AIE with RO25–6981 [F_(1,16)_ = 7.96, p = 0.012, Fig. 2, left panel]. In addition, there was a significant main effect of RO25–6981 [F_(1,16)_ = 107.26, p < 0.001] and a nearly significant effect of AIE [F_(1,16)_ = 4.38, p = 0.053]. Given the significant interaction between AIE and RO25–6981 we performed a simple main effects analysis which indicated that eEPSC amplitude was significantly greater in slices from AIE animals than in those from AIW animals [t_(16)_ = 2.53, p = 0.01] prior to application of RO25–6981 (Fig. 2, left panel, baseline), but not after [t_(16)_ = 0.11, p = 0.46]. Application of RO25–6981 reduced NMDA current amplitude in slices from both AIW [t_(9)_ = 6.2, p < 0.001] and AIE [t_(7)_ = 8.02, p < 0.001] animals (Fig. 2, left panel).

Using gabapentin pre-incubated slices to evaluate our second hypothesis, RO25–6981 was not found to differentially reduce eEPSC amplitude in slices from AIE animals compared to those from AIW animals (Fig. 2, right panel). Gabapentin pre-incubation also eliminated the AIE x RO25–6981 interaction that we observed in slices that were not pre-incubated with gabapentin, consistent with our hypotheses. In gabapentin pre-incubated slices, there was a significant effect of RO25-6981 [F_(1,16)_ = 47.41, p < 0.001], but no effect of AIE [F_(1,16)_ = 2.75, p = 0.12) and no AIE x RO25-6981 interaction [F_(1,16)_ = 0.73, p = 0.41, Fig. 2, right panel].

**Figure 3:**
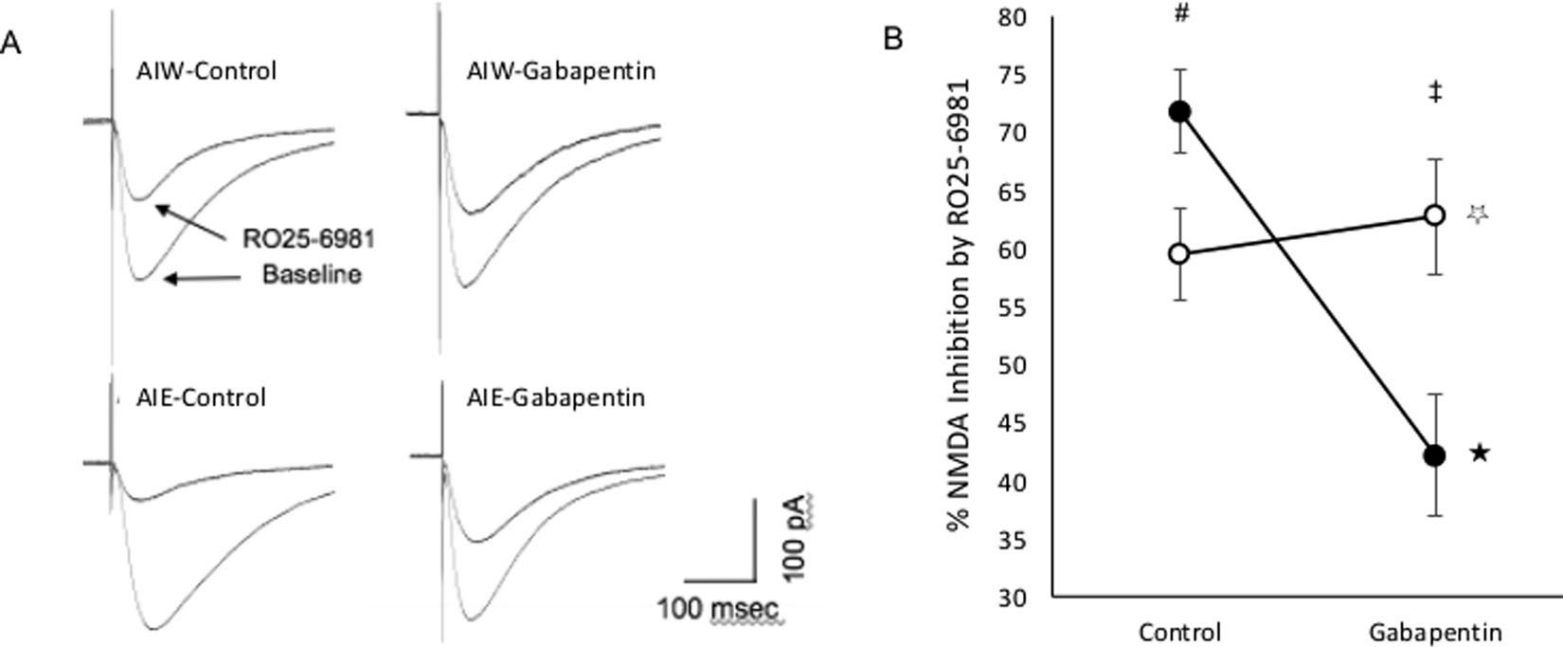
Effect of AIE, and Gabapentin Slice Pre-Incubation on RO25–6981-induced Inhibition of NMDA Receptor-Mediated Currents. A. Representative eEPSCs recorded in cells from control (AIW) animals (upper panels) and AIE-exposed (AIE) animals (lower panels) after slices were pre-incubated in normal aCSF (Control - left panels) or aCSF containing 30 μM gabapentin (Gabapentin - right panels). RO25–6981 consistently reduced current amplitude, but did so more efficaciously in cells from slices that were not pre-incubated in gabapentin. B. Mean (±SEM) percent reduction of NMDA receptor-mediated current amplitudes by RO25–6981, recorded in CA1 pyramidal cells from AIE-exposed animals (closed circles) compared to controls (open circles), after pre-incubation in aCSF (Control) or aCSF containing 30 μM gabapentin (Gabapentin). RO25–6981 induced inhibition was greater after AIE than after AIW when slices were pre-incubated in aCSF [right panel (Control, #): simple main effect, p = 0.02]. Conversely, RO25–6981 induced inhibition was greater in AIW than in AIE when slices were pre-incubated in aCSF+ gabapentin [right panel (Gabapentin, ‡): simple main effect, p = 0.006]. Moreover, gabapentin pre-incubation significantly attenuated RO25–6981-induced inhibition of NMDA current amplitudes in slices from AIE animals [right panel (closed circles,★): simple main effect, p = 0.001] but not in those from AIW animals [right panel (open circles, ☆), simple main effect, p = 0.28].

To further evaluate the effect of gabapentin pre-incubation, we calculated the percent decrease in eEPSC amplitude induced by RO25-6981 and assessed the relative and combined effects of AIE and gabapentin pre-incubation. Consistent with our hypotheses, gabapentin pre-incubation attenuated the RO25-6981 induced decline in eEPSC amplitude in slices from AIE animals but not in those from AIW animals (Fig. 3A,B). Analyses of variance revealed a significant main effect of gabapentin pre-incubation [F_(1,16)_ = 10.78, p = 0.005] and a significant interaction [F_(1,16)_ = 16.83, p = 0.001), but no effect of AIE (F_(1,16)_ = 0.68, p = 0.21). Simple main effects revealed greater inhibition of current amplitudes by RO25-6981 in cells from AIE animals than in those from AIW animals in the absence of gabapentin pre-incubation [t_(16)_ = −2.25, p = 0.02; Fig. 3, right panel, Control (#)]. In slices that had been pre-incubated with gabapentin RO25-6981 produced more current inhibition in cells from AIW animals than in those from AIE animals [t_(16)_ = 2.83, p = 0.006; Fig. 3, right panel, Gabapentin (‡)]. More importantly, gabapentin pre-incubation attenuated the RO25-6981-induced decline in eEPSC amplitude in slices from AIE animals [t_(7)_ = 5.03, p = 0.001; Fig. 3, right panel, closed circles ★)] but not in those from AIW animals [t_(9)_ = 0.61, p = 0.28; Fig. 3, right panel, open circles (☆)].

## Discussion

The principal findings in this report are that, 1) AIE caused both an increase in the amplitude of NMDA receptor-mediated eEPSCs in hippocampal CA1 pyramidal cells and an increase in the proportion of those currents that was driven by GluN2B receptors, and 2) both of those AIE effects were reversed by pre-incubation of hippocampal slices in aCSF containing gabapentin. These findings suggest that an increase in NMDA receptor-mediated synaptic function in the hippocampal formation after AIE may underlie the propensity toward excitatory synaptic plasticity^15^ and neuronal loss^16^ that we have observed previously in hippocampal area CA1. The reversal of AIE-induced increases in NMDA eEPSC amplitude by gabapentin is particularly noteworthy because it suggests that a known and well-tolerated agent in current clinical usage may reverse some of the enduring effects of AIE on neural function.

The present finding of upregulated NMDA receptor-mediated synaptic function after AIE, and increased proportional drive of those currents by GluN2B receptors, may be of mechanistic importance for understanding ethanol-induced impairment of hippocampal function. For example, GluN2B receptors have been shown to drive a form of LTP that is induced with low intensity stimulus trains (as was the LTP we observed to be enhanced after AIE in Risher *et al*.^15^)^22^, and a recent study has shown enhancement of LTP induction in CA1 in an experimental model of temporal lobe epileptogenesis^23^, very much like what we have observed after AIE^15^. The enhancement of LTP induction, in the context of epileptogenesis, was blocked by the GluN2B antagonism but not by GluN2A antagonism, and the proportion of NMDAR-mediated current driven by the GluN2B subtype was increased relative to that carried by the GluN2A subtype in CA1 pyramidal neurons from the epileptogenic animals^23^. Thus, the present findings are consistent with the hypothesis that AIE induces susceptibility to hyperexcitability in hippocampal circuits, which may be driven by an increase in NMDA receptor-mediated synaptic activity driven by a shift toward GluN2B mediation.

GluN2B-containing receptors tend to be localized extrasynaptically, whereas GluN2A receptors are more prevalent in synaptic regions, though both subtypes have been shown to migrate between synaptic and extrasynaptic loci^24,25^. Calcium currents carried through GluN2A receptor complexes induce gene expression of cAMP response element binding protein (CREB)-evoked brain derived neurotropic factor (BDNF), which is neuroprotective^18^. In contrast, Ca^2+^ currents carried by GluN2B receptors inhibit BDNF expression^26^. These findings indicate that, while GluN2A and GluN2B receptors overlap in terms of both localization and function, the former may contribute more to anti-apoptotic activity, whereas the latter appear to contribute more to processes that could result in neurotoxicity and neuronal death^27^. Thus, the shift toward a greater GluN2B drive of NMDA currents after AIE could predispose neurons to excitotoxic liability. One limitation of the present data set is that our analysis was restricted to the assessment of one specific NMDA receptor subtype (GluN2B). The assessment of antagonists for other subtypes, notably GluN2A (such as TCN 201) would have allowed a more fine-grained assessment of possible AIE effects on receptor subtype interactions. However, we chose to address GluN2B antagonism exclusively in an effort to limit the duration of recording sessions and assure the health of cells and integrity of recorded currents. Clearly, the present findings represent a first step in the analysis of NMDA receptor subtype alterations after AIE, and the gabapentin-induced reversals that we observed will help to guide future studies.

AIE may also induce aberrant excitatory synaptogenesis. As noted above, astrocyte-released TSPs are synaptogenic and strongly promote the formation of excitatory synapses by virtue of their interaction with the neuronal α2δ−1 receptor^17,28^. Both synaptogenic TSPs and the α2δ−1 receptor are upregulated in hippocampal area CA1 in adulthood after AIE^16^, suggesting aberrant elevations of excitatory synaptogenesis that which could contribute to the LTP propensity and cell loss we have observed in that region^15,16^ through an excitotoxic mechanism. If an AIE-induced, TSP mediated increase in excitatory synaptogenesis drives the increased amplitude of NMDA receptor mediated currents observed in the present study, the reversal of that increase by one hour of gabapentin pre-incubation may provide clues as to the mechanisms underlying those effects. In one sense it seems unlikely that one hour of gabapentin pre-incubation would reverse increased receptor expression. However, in addition to synaptogenic effects, TSPs are also involved in the maintenance and function of existing synapses^17,28^. Thus, it could be that a transient gabapentin block of TSP binding at the α2δ−1 site could downregulate the function of NMDA receptors for a period of time sufficient to affect the observations in the present study. Whether or not this is the case, the findings raise two important questions related to the duration of effect of a single gabapentin exposure on AIE-induced changes and whether gabapentin treatment during AIE could prevent the changes that result in the present findings. Indeed, a study designed to assess the effects of gabapentin co-administration during the period of AIE exposure would address whether gabapentin can prevent the enduring effects of AIE on NMDA function. The present study was designed to assess reversal or amelioration (rather than prevention) of AIE effects because those questions seem more translationally relevant from a treatment perspective. Still, the issue of prevention is important and will be addressed in future studies.

The present finding that gabapentin reverses AIE-induced enhancement of NMDA currents and the shift toward their drive by GluN2B receptors is particularly important from a translational standpoint. Gabapentin (Neurontin) is a well-tolerated medication in long-standing clinical use for the treatment of neuropathic pain and restless leg syndrome, and as an adjunctive treatment for partial onset seizures and bipolar disorder. Its anticonvulsant effect is consistent with our observation that it reverses the increase in NMDA receptor-mediated neuronal excitability and suggests that it could be an agent with efficacy against some of the enduring neural and cognitive effects of AIE. From a mechanistic standpoint these results suggest that enduring effect of AIE on hippocampal function may be mediated, at least in part, by aberrant astrocyte activation and the interaction of astrocyte-released TSPs with neuronal α2δ−1 receptors.

Taken together, these findings identify a previously unknown upregulation of NMDA receptor-mediated function in hippocampal area CA1 in adulthood after adolescent ethanol exposure, and a shift in the balance of NMDA receptor subtypes that drive those currents. The reversal of those effects by gabapentin suggests a novel, astrocyte-mediated mechanism underlying those effects and a new translational target for possible amelioration of the enduring effects of ethanol on neural and cognitive function.
